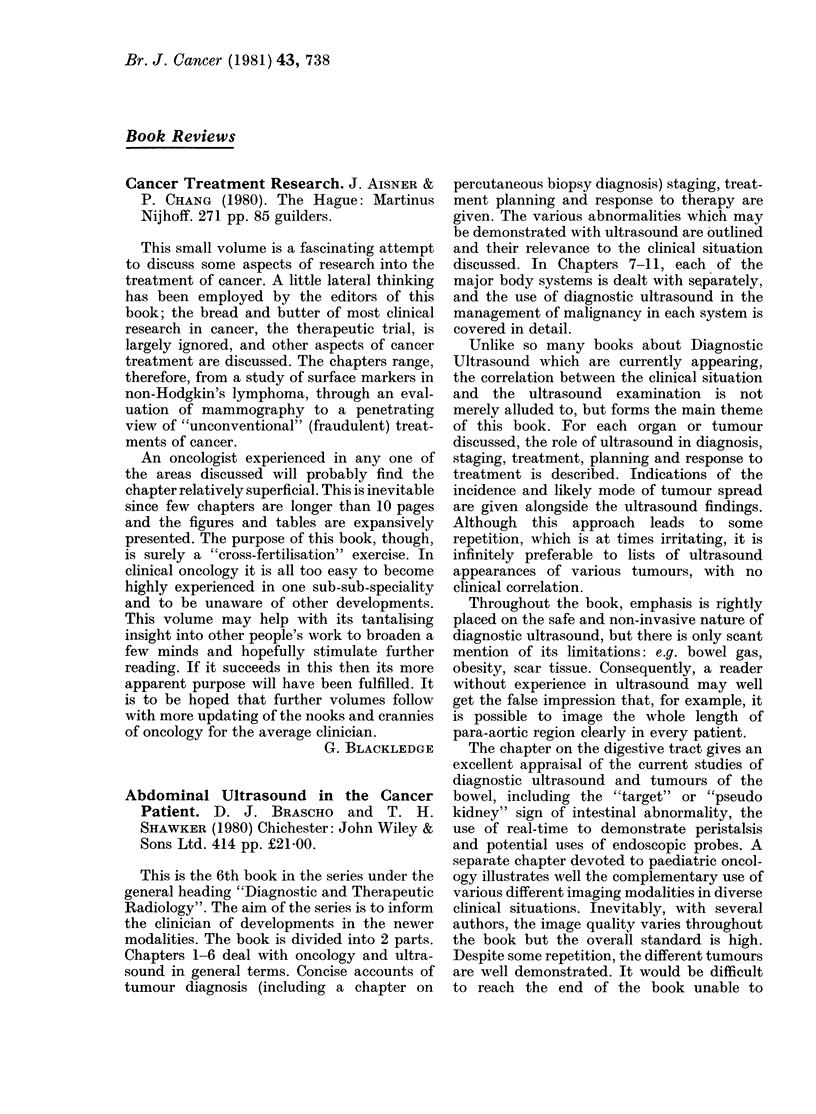# Cancer Treatment Research

**Published:** 1981-05

**Authors:** G. Blackledge


*Br. J. Cancer* (1981) **43**, 738

### *Book Reviews*

**Cancer Treatment Research.** J. AISNER & P. CHANG (1980). The Hague: Martinus Nijhoff. 271 pp. 85 guilders.

This small volume is a fascinating attempt to discuss some aspects of research into the treatment of cancer. A little lateral thinking has been employed by the editors of this book; the bread and butter of most clinical research in cancer, the therapeutic trial, is largely ignored, and other aspects of cancer treatment are discussed. The chapters range, therefore, from a study of surface markers in non-Hodgkin's lymphoma, through an evaluation of mammography to a penetrating view of "unconventional" (fraudulent) treatments of cancer.

An oncologist experienced in any one of the areas discussed will probably find the chapter relatively superficial. This is inevitable since few chapters are longer than 10 pages and the figures and tables are expansively presented. The purpose of this book, though, is surely a "cross-fertilisation" exercise. In clinical oncology it is all too easy to become highly experienced in one sub-sub-speciality and to be unaware of other developments. This volume may help with its tantalising insight into other people's work to broaden a few minds and hopefully stimulate further reading. If it succeeds in this then its more apparent purpose will have been fulfilled. It is to be hoped that further volumes follow with more updating of the nooks and crannies of oncology for the average clinician.

G. BLACKLEDGE